# Checklist of Iranian Encyrtids (Hymenoptera: Chalcidoidea) with Descriptions of New Species

**DOI:** 10.1673/031.010.6801

**Published:** 2010-06-18

**Authors:** Majid Fallahzadeh, George Japoshvili

**Affiliations:** ^1^ Department of Entomology, Islamic Azad University, Jahrom Branch, Fars, Iran; ^2^ Department of Plant Protection, Faculty of Agriculture, Suleyman Demirel University, Isparta, Turkey; ^3^ Entomology and Biocontrol Research Centre, Ilia State University, Tbilisi, Georgia

**Keywords:** New species, Hymenoptera, Encyrtidae, Distribution, Iran

## Abstract

A list of Iranian Encyrtidae (Hymenoptera: Chalcidoidea) is given for the first time. It includes 93 species representing 32 genera. Host information from Iran and distributional data are also provided. Three genera and 7 species are first recorded from Iran. New host records are provided for three species. Two new species, *Gyranusoidea iranica* sp. n. and *Microterys iranicus* sp. n., are described and diagnostic characters are provided for them.

## Introduction

The Encyrtidae is the most speciose group of parasitoids attacking scale and psyllid insects. Members of the family are important in biological control. More than 400 encyrtid species have been used or are used today for suppression of various crop pests (Japoshvili and Noyes 2006). There are more then 1270 described species of encyrtids in the Palaearctic Region (Trjapitzin 1989; Yasnosh and Japoshvili 1999; Japoshvili 2005, 2007a, b; Japoshvili and Karaca 2003; Japoshvili and Abrantes 2006; Japoshvili and Noyes 2005, 2006).

The encyrtid fauna in Iran has been a subject of special investigations only in the last few years.The first published records of Iranian Encyrtidae were those by Kiriukhin (1947), who reported some Encyrtid parasitoids of scale insects. Several species were subsequently recorded by Farahbakhsh (1961), Chodjai (1968), Davatchi and Chodjai (1969), OILB (1971), Herting (1971, 1972), Chodjai (1976, 1989) and Radjabi (1986) Papers by Asadeh and Mossadegh (1991, 1993), Ebrahimi (1993), and Yazdani and Mehrnejad (1993) contain some faunistical data on Iranian Encyrtidae. Modares Awal (1997) cited 31 species of encyrtids in his list of agricultural pests and their natural enemies in Iran. Ferrière (1961), Myartseva and Sugonjaev (1977), Myartseva (1980), Xu and Lotfalizadeh (2000), Noyes and Fallahzadeh (2005) and Manickavasagam, Mehrnejad (2008) described new species from Iran. Noyes (2009) in his database lists 42 species from Iran. In recent years some faunistical studies have been conducted on Iranian Encyrtidae and currently the number of species of this family recorded in Iran has increased to about 84 (Lotfalizadeh and Ebrahimi 2001; Davoodi et al. 2004 a, b, c, d; Fallahzadeh and Hesami 2004; Japoshvili and Noyes 2005; Fallahzadeh et al. 2006a, b, 2007 2008; Talebi et al. 2008). This is a modest result, given that the area of Iran is almost twice that of Turkey and Transcaucasus combined, where 307 species were recorded (Japoshvili, Nayes 2005).

Although many studies have been conducted on the Encyrtidae of Iran, their results were scattered in different publications and have never been summarized. The present list was compiled to provide a reference for future studies on this family in Iran. It includes insect hosts and associated host plants from Iran.

Ninety three species of Encyrtidae belonging to 32 genera are currently known from Iran. All references about Iranian Encyrtids are summarized and synonyms recorded from Iran are provided, as well as host information and distributional data. Three genera and seven species are first recorded from Iran. Additionally, new hosts are provided for three species. Two new species, *Gyranusoidea iranica* Japoshvili and Fallahzadeh sp. nov. and *Microterys iranicus* Japoshvili and Fallahzadeh sp. nov. are described.

## Material and Methods

Material was collected from 2005 to 2007 in different parts of Iran, and voucher specimens for new records and new species are deposited in the Insect collection of Entomology and Biocontrol Research centre, Ilia State University, Tbilisi, Georgia. All available literature sources were summarized for the first time as well. Taxonomic concepts mostly follow Trjapitzin (1989), Gibson et al. (1997) and Noyes (2009). Synonyms, worldwide distributions, and host information, from outside Iran are not included as most of these data are provided in the Universal Chalcidoidea Database (Noyes 2009). All measurements were done using Hirox digital microscope KH-7700. Description and terminology follows Noyes (2009).

## Results and Discussion

The list of Iranian Encyrtidae now contains 93 species belonging to 32 genera. Three genera not previously recorded from Iran are: *Anicetus* Howard, 1896, *Gyranusoidea *Compere, 1947 and *Paranathrix* Myartseva, 1980. Seven species that are marked with an asterisk are newly recorded for the country. Additionally three species that are marked with double asterisks have new host records. Two new species: *Gyranusoidea iranica* and *Microterys iranicus* are described. The complete list of Encyrtid wasps of Iran follows. Iran is a large country incorporating various geographical regions and climates and we expect that many species remain to be discovered. More studies should be conducted on this important insect group in Iran.

**Genus *Ageniaspis* Dahlbom, 1857 *Ageniaspis fuscicollis* (Dalman, 1820)**
Synonym in Iranian literature: *Encyrtus fuscicollis* Dalman, 1820 (Radjabi 1986; Safaralizadeh 1984; Moddarres Awal 1997).
Host: *Hyponomeuta malinellus* [*Yponomeuta malinellus* Zeller] (Lepidoptera: Yponomeutidae), *H. padellus *[*Y. padella* (L.)] on fruit tree (Rosaceae), *H. rolellus* [*Y. rorrella* (Hübner)] on *Salix* (Salicaceae) (Esmaili 1983; Safaralizadeh 1984; Radjabi 1986; Moddarres Awal 1997), *Y. malinellus* on *Malus* sp. (Rosaceae) (Haeselbarth 1983).
Iranian records: Azerbaijan, Hamadan, Kurdestan, Markazi, Zanjan, Tehran, Qazvin provinces (Esmaili 1983; Safaralizadeh 1984; Radjabi 1986; Moddarres Awal 1997), Iran (Trjapitzin 1978; Haeselbarth 1983; Trjapitzin 1989).

***Ageniaspis testaceipes* (Ratzeburg, 1848)**
Synonym in Iranian literature: *Ageniaspis (Holcothorax) testaceipes* (Ratzeburg 1848) (Chodjai 1968; Davatchi and Chodjai 1969; Chodjai 1989; Behdad 1991; Moddarres Awal 1997).
Host: *Lithocolletis platani* [*Phyllonorycter platani* (Staudinger)] (Lepidoptera: Gracillariidae) on *Platanus orientalis* L. (Platanaceae) (Chodjai 1968; Chodjai 1989; Davatchi and Chodjai 1969; Trjapitzin 1989).
Iranian records: Tehran province (Chodjai 1968; Davatchi and Chodjai 1969; Chodjai 1989; Behdad 1991; Moddarres Awal 1997), Iran (Trjapitzin 1989).

**Genus *Anagyrus* Howard, 1896 *Anagyrus agraensis* Saraswat, 1975**
Synonym in Iranian literature: *Anagyrus indicus* Shafee, Alam and Agarwal, 1975 (Awal, 1997).
Hosts: *Nipaecoccus viridis* (Newstead) (Hemiptera: Pseudococcidae) on *Citrus* (Rutaceae) and *Morus alba* L. (Moraceae) (Asadeh and Mossadegh 1991, 1993; Noyes and Hayat 1994; Baniameri and Mossadegh 1998a, 1998b; Novin et al. 2000; Hesami and Fallahzadeh 2005), *Maconellicoccus hirsutus* (Green) (Hemiptera: Pseudococcidae) on *M. alba* L. (Moraceae) (Fallahzadeh and Hesami 2004; Fallahzadeh et al. 2007).
Iranian records: Khuzestan province (Asadeh and Mossadegh 1991, 1993; Noyes and Hayat 1994; Moddarres Awal 1997; Baniameri and Mossadegh 1998a, 1998b; Novin et al. 2000), Tehran province (Noyes and Hayat 1994), Fars province (Fallahzadeh and Hesami 2004; Hesami and Fallahzadeh 2005; Fallahzadeh et al. 2007).

***Anagyrus aligarhensis* Agarwal and Alam, 1959**
Synonym in Iranian literature: *Anagyrus diversicornis* Mercet, 1921 (Asadeh and Mosadegh 1991; 1993; Noyes and Hayat 1994; Novin et al. 2000; Hesami and Fallahzadeh 2004, 2005).
Host: *N. viridis* (Newstead) (Hemiptera: Pseudococcidae) on *Citrus* (Rutaceae) and *M. alba* L. (Moraceae) (Asadeh and Mossadegh 1991, 1993; Noyes and Hayat 1994; Novin et al. 2000; Hesami and Fallahzadeh 2004, 2005).
Iranian records: Khuzestan province (Asadeh and Mosadegh 1991, 1993; Novin et al. 2000), Tehran province (Noyes and Hayat 1994), Fars province (Hesami and Fallahzadeh 2004; 2005).

***Anagyrus dactylopii* (Howard, 1898)**
Hosts: *N. viridis* (Newstead) (Hemiptera: Pseudococcidae) on *Citrus* (Rutaceae) and *Morus alba* L. (Moraceae) (Asadeh and Mossadegh 1991, 1993; Noyes and Hayat 1994; Baniameri and Mossadegh 1998a; 1998b; Novin et al. 2000; Dezhakam and Soleyman Nejadian 2000; Hesami and Fallahzadeh 2005), *Planococcus citri* (Rissio) on *Citrus* (Maafi et al. 1998); *M. hirsutus* (Green) (Hemiptera: Pseudococcidae) on *M. alba* L. (Moraceae) (Fallahzadeh and Hesami 2004; Fallahzadeh et al. 2007).
Iranian records: Khuzestan province (Asadeh and Mossadegh 1991, 1993; Noyes and Hayat 1994; Mossadegh and Baniameri 1996; Baniameri and Mossadegh 1998a; Novin et al. 2000; Dezhakam and Soleyman Nejadian 2000), Tehran province (Noyes and Hayat 1994), Mazandaran province (Maafi et al. 1998), Fars province (Fallahzadeh and Hesami 2004; Hesami and Fallahzadeh 2005; Fallahzadeh et al. 2007).

***Anagyrus kamali* Moursi, 1948**
Host: *M. hirsutus* (Green) (Hemiptera: Pseudococcidae) on *M. alba* L. (Moraceae) (Fallahzadeh et al. 2007).
Iranian record: Fars province (Fallahzadeh et al. 2007).

***Anagyrus matritensis* (Mercet, 1921)**
Synonym in Iranian literature: *Anagyrus orbitalis* (Ruschka 1923) (Fallahzadeh et al. 2006b).
Host: *Peliococcus kimmericus *(Kiritshenko) (Hemiptera: Pseudococcidae) on *Lactuca serriola* L. (Asteraceae) (Fallahzadeh et al. 2006b). Iranian record: Fars province (Fallahzadeh et al. 2006b).

***Anagyrus mirzai* Agarwal and Alam, 1959**
Host: *N. viridis* (Newstead) (Hemiptera: Pseudococcidae) on *Citrus* (Rutaceae) (Novin 2000, Hesami and Fallahzadeh 2005), *M. hirsutus* (Green) (Hemiptera: Pseudococcidae) on *M. alba* L. (Moraceae) (Fallahzadeh and Hesami 2004; Fallahzadeh et al. 2007).
Iranian records: Khuzestan province (Novin 2000), Fars province (Fallahzadeh and Hesami 2004; Hesami and Fallahzadeh 2005; Fallahzadeh et al. 2007).

***Anagyrus pseudococci* (Girault, 1915)**
Hosts: *P. citri* (Risso) (Hemiptera: Pseudococcidae) on *Ficus carica* L. (Moraceae) (Chodjai 1968, 1976, 1989), *P. citi* (Hemiptera: Pseudococcidae) and *Marietta picta* (Andre) on *Vitis vinifera* L. (Vitaceae) (Hymenoptera, Aphelinidae) (OILB, 1971), *Pseudococcus filamentosus* [*Nipaecoccus viridis* (Newstead)] (Hemiptera, Pseudococcidae) on *Citrus* (Khalaf and Aberoumand 1986), *Planococcus vovae* (Nasonov) on cypress tree (Cupressaceae) (Hemiptera, Pseudococcidae) (Xu and Lotfalizadeh 2000; Lotfalizadeh and Ahmadi 2000; Talebi et al. 2008); *N. viridis* on *Citrus* (Rutaceae) (Hesami and Fallahzadeh 2004, 2005) *M. hirsutus* (Green) (Hemiptera: Pseudococcidae) on *M. alba* L. (Moraceae) (Fallahzadeh and Hesami 2004; Fallahzadeh et al. 2007).
Iranian records: Tehran province (Chodjai 1968, 1976, 1989; Moddarres Awal1994; Talebi et al. 2008) Fars province (Khalaf and Aberoumand 1986; Moddarres Awal 1997; Xu and Lotfalizadeh 2000; Lotfalizadeh and Ahmadi 2000; Fallahzadeh and Hesami 2004; Hesami and Fallahzadeh 2004; Hesami and Fallahzadeh 2005; Fallahzadeh et al. 2007); Iran (OILB 1971).

***Anagyrus schoenherri* (Westwood, 1837)**
Hosts: *Eulecanium coryli* [*Eulecanium tiliae* (Linnaeus)] and *Pulvinaria betulae *[*Pulvinaria vitis* (Linnaeus)] (Hemiptera: Coccidae) on rosaceous fruit trees (Rosaceae) (Radjabi 1989); *P. vitis* (Herting 1972), *Phenacoccus aceris* (Signoret) (Hemiptera: Pseudococcidae) (Kiriukhin 1947; Herting 1972; Trjapitzin 1978, 1989). Iranian records: Iran (Kiriukhin 1947; Herting 1972; Trjapitzin 1978, 1989; Noyes and Hayat 1994), Caspian Sea area, Markazi, Tehran provinces (Farahbakhsh 1961, Radjabi 1989; Moddarres Awal 1997).

Genus *Anicetus* Howard, 1896
* *Anicetus italicus* (Masi, 1917)
Material examined: 4♀, 2♂, Estahban, Fars province, ex *Ceroplastes rusci* L. (Hemiptera: Coccidae) on *Ficus carica* L. (Moraceae), 28.IX.2007, M. Fallahzadeh. Voucher specimens housed in EBRC.

**Genus *Blastothrix* Mayr, 1876 *Blastothrix brittanica* Girault, 1917**
Hosts: *Diaspidiotus prunorum* (Laing) (Hemiptera: Diaspididae) and *E. coryli* [*Eulecanium tiliae* (Linnaeus)] (Hemiptera: Coccidae) on rosaceous fruit trees (Rosaceae) (Dastghyb Beheshti et al. 1988; Radjabi 1989; Behdad 1991; Moddarres Awal 1997).
Iranian records: Isfahan province (Dastghyb Beheshti et al. 1988; Radjabi 1989; Behdad 1991; Moddarres Awal 1997).

***Blastothrix sericea* (Dalman, 1820)**
Hosts: *Sphaerolecanium prunastri* (Boyer de Fonscolombe), *Eulecanium coryli* [*E. tiliae* (Linnaeus)], *E. tiliae* (Hemiptera: Coccidae) on rosaceous fruit trees (Rosaceae) (Behdad and Barouti 1978; Farahbakhsh 1961; Radjabi 1989; Moddarres Awal 1997); *Anapulvinaria pistaciae* (Bodenheimer) (Hemiptera: Coccidae) on *Pistacia vera* L. (Anacardiaceae) (Yazdani and Rajabi 1993), *E. tiliae* on quince tree, apple tree (Rosaceae); *E. coryli* [*E. tiliae*] on cherry tree, apple tree, plum tree, apricot tree (Rosaceae) *S. prunastri* on plum tree (Rosaceae) (Davoodi et al. 2004d).
Iranian records: Isfahan, Tehran and Markazi provinces (Behdad and Barouti 1978; Farahbakhsh 1961; Radjabi 1989; Moddarres Awal 1997) Kerman provine (Yazdani and Rajabi 1993) Tehran and Gilan provinces (Davoodi et al. 2004d).

***Blastothrix ilicicola* Mercet, 1921**
Host: *E. coryli* [*E. tiliae* (Linnaeus)] (Hemiptera: Coccidae) on quince tree (Rosaceae) (Ebrahimi 1993).
Iranian record: Chaharmahal-Bakhtiyari province (Ebrahimi 1993).

***Blastothrix hungarica* Erdös, 1959**
Host: *Eulecanium* sp. (Hemiptera: Coccidae) on *Ficus carica* L. (Moraceae) (Hesami et al. 2008)
Iranian record: Fars province (Hesami et al. 2008).

***Blastothrix turanica* Sugonjaev, 1964**
Host: Unknown from Iran.
Iranian record: Iran (Trjapitzin 1989).

**Genus *Cerapterocerus* Westwood, 1833 *Cerapterocerus mirabilis* Westwood, 1833**
Hosts: *Aonidiella orientalis* (Newstead) (Hemiptera: Diaspididae) on *Citrus* (Rutaceae) (Chodjai 1963; Chodjai 1989; Davatchi and Chodjai 1969; Behdad 1991; Moddarres Awal 1997), Hyperparasitoid of *S. prunastri* (Boyer de Fonscolombe) (Hemiptera: Coccidae) (Davoodi et al. 2004d).
Iranian records: Kerman province (Chodjai 1963; Davatchi and Chodjai 1969; Chodjai 1989; Behdad 1991; Moddarres Awal 1997), Tehran province (Davoodi et al. 2004d).

**Genus *Cheiloneurus* Westwood, 1833 *Cheiloneurus ceroplastis* Ishii, 1923**
Hosts: *Chrysoperla carnea* Stephens and *Suarias fedtchenkoi* (McLachlan) (Neuroptera: Chrysopidae) on cypress tree (Cupressaceae) (Xu and Lotfalizadeh 2000; Lotfalizadeh and Ahmadi 2000; Lotfalizadeh 2000a).
Iranian records: Fars province (Xu and Lotfalizadeh 2000; Lotfalizadeh and Ahmadi 2000; Lotfalizadeh 2000a).

***Cheiloneurus claviger* Thomson, 1876**
Host: *S. prunastri* (Boyer de Fonscolombe) (Hemiptera: Cocidae) on prunaceous trees (Talebi et al. 2009).
Iranian records: Khorasan-e-Razavi province (Talebi et al. 2009).
Material examined: 1♀, Mian-Jangle, Fasa, Fars province, 22°12′N, 53°23′E, 1680m, ex *S. prunastri* (Boyer de Fonscolombe) (Hemiptera: Coccidae) on *Prunus scoparia* (Spach) (Rosaceae), 5.VI.2007, M. Fallahzadeh. Voucher specimens housed in EBRC.

***Cheiloneurus kollari* (Mayer, 1876)**
Host: *P. kimmericus* (Kiritshenko) (Hemiptera: Pseudococcidae) on *Lactuca serriola* L. (Asteraceae) (Fallahzadeh et al. 2006b).
Iranian records: Fars province (Fallahzadeh et al. 2006b).

***Cheiloneurus paralia* (Walker, 1837)**
Host: *P. kimmericus* (Kiritshenko) (Hemiptera: Pseudococcidae) on *L. serriola* L. (Asteraceae) (Fallahzadeh et al. 2006b). Iranian records: Fars province (Fallahzadeh et al. 2006b).

***Cheiloneurus pistaciae* Manickavasagam and Mehrnejad, 2008**
Hosts: A facultative hyperparasitoid of pistachio twig borer moth, *Kermania pistaciella* (Lepidoptera: Tineidae), via *Chelonus kermakiae* (Hymenoptera: Braconidae) on *P. vera* L. (Anacardiacea) (Manickavasagam et al. 2008).
Iranian records: Kerman province (Manickavasagam et al. 2008).

**Genus *Choreia* Westwood, 1833 *Choreia maculata* (Hoffer, 1954)**
Host: Unknown from Iran.
Iranian records: Iran (Trjapitzin 1978, 1989).

**Genus *Coccidoxenoides* Girault, 1915 *Coccidoxenoides perminutus* Girault, 1915**
Host: *P. vovae* (Nasonov) (Hemiptera: Pseudococcidae) on cypress tree (Cupressaceae) (Talebi et al. 2008).
Iranian record: Tehran province (Talebi et al. 2008).

**Genus *Comperiella* Howard, 1906 *Comperiella bifasciata* Howard, 1906**
Host: *A. orientalis* (Newstead) (Hemiptera: Diaspididae) on *Citrus* (Rutaceae) (Esmaili 1983; Moddarres Awal 1997).
Iranian records: Hormozgan province (Esmaili 1983; Moddarres Awal 1997).

***Comperiella lemniscata* Compere and Annecke, 1961**
Host: *A. orientalis* (Newstead) (Hemiptera: Diaspididae) on *Citrus* (Rutaceae) (Lachinani and Ahmadi 1993; Prinsloo 1996).
Iranian records: Fars province (Lachinani and Ahmadi 1993); Iran (Prinsloo 1996).

**Genus *Copidosoma* Ratzeburg, 1844 *Copidosoma pistacinellae* Hoffer, 1970**
Host: *Recurvaria pistacinella* Danilevsky (Lepidoptera: Gelechiidae) on *P. vera* L. (Anacardiaceae) (Hoffer 1970; Trjapitzin 1989).
Iranian records: Iran (Hoffer 1970; Trjapitzin 1989).

***Copidosoma varicorne* (Nees, 1834)**
Synonyms in Iranian literature: *Encyrtus varicornis* Nees, 1834, *Paralitomastix varicornis* (Nees 1834) (Chodjai 1968; Chodjai 1989; Sabzevari 1968; Esmaili 1983; Radjabi 1986; Behdad 1991; Moddarres Awal 1997).
Host: *Anarsia lineatella* (Zeller) (Lepidoptera: Gelechiidae) on Roseous fruit trees (Rosaceae) (Chodjai 1968; Chodjai 1989; Sabzevari 1968; Esmaili 1983; Radjabi 1986; Behdad 1991; Moddarres Awal 1997; OILB 1971).
Iranian records: Azerbaijan, Markazi, Tehran provinces (Chodjai 1968; Chodjai 1989; Sabzevari 1968; Esmaili 1983; Radjabi 1986; Behdad 1991; Moddarres Awal 1997), Iran (OILB 1971; Trjapitzin 1989; Guerrieri and Noyes 2005).

**Genus *Discodes* Förster, 1856 *Discodes coccophagus* (Ratzeburg, 1848)**
Host: *S. prunastri* (Boyer de Fonscolombe) (Hemiptera: Cocidae) on prunaceous trees (Talebi et al. 2009).
Iranian records: Khorasan-e-Razavi province (Talebi et al. 2009).
Material examined: 6♀, 2♂, Mian-Jangle, Fasa, Fars province, 22°12′N, 53°23′E, 1680m, ex *S. prunastri* (Boyer de Fonscolombe) (Hemiptera: Coccidae) on *Prunus scoparia* (Spach) (Rosaceae), 5.VI.2007, M. Fallahzadeh. Voucher specimens housed in EBRC.

**Genus *Dusmetia* Mercet, 1921 *Dusmetia fuscipennis* (Noyes and Hayat, 1984)**
Host: *P. vovae* (Nasonov) (Hemiptera: Pseudococcidae) on cypress tree (Cupressaceae) (Xu and Lotfalizadeh 2000; Lotfalizadeh and Ahmadi 2000).
Iranian records: Fars province (Xu and Lotfalizadeh 2000; Lotfalizadeh and Ahmadi 2000).

**Genus *Encyrtus* Latreille, 1809 *Encyrtus aurantii* (Geoffroy, 1785)**
Synonym in Iranian literature: *Encyrtus lecaniorum* (Mayr, 1876) (Farahbakhsh 1961; Moddarres Awal 1997; Esfandiari et al. 2005).
Hosts: *E. coryli* [*E. tiliae* (Linnaeus)] (Farahbakhsh 1961), *E. tiliae* (Hemiptera: Coccidae) on elm tree (Ulmaceae), *E. coryli* [*E. tiliae*] on apple tree (Rosaceae), *Coccus hesperidum* Linnaeus (Hemiptera: Coccidae) on *Robinia* (Papilionaceae); *Morus* (Moraceae), *Convolvulus arvensis* L. (Convolvulaceae), *S. prunastri* (Boyer de Fonscolombe) on prune tree (Rosaceae) (Davoodi et al. 2003c; Davoodi et al. 2004d), *S. prunastri* (Boyer de Fonscolombe) on *Amygdalus* (Rosaceae) Esfandiari et al. 2005).
Iranian records: Gilan, Mazandaran provinces (Farahbakhsh 1961; Moddarres Awal 1997), Gilan and Tehran provinces (Davoodi et al. 2003c, 2004d), Chaharmahal-Bakhtiyari province (Esfandiari et al. 2005), Iran (Trjapitzin, Myartseva 2004).

***Encyrtus infidus* (Rossi, 1790)**
Synonym in Iranian literature: *Encyrtus scutellatus* (Swederus, 1795) (Esfandiari 2002; Esfandiari et al. 2005).
Host: *S. prunastri* (Boyer de Fonscolombe) on *Amygdalus* (Rosaceae) (Esfandiari 2002; Esfandiari et al. 2005).
Iranian record: Chaharmahal-Bakhtiyari province (Esfandiari 2002; Esfandiari et al. 2005).

***Encyrtus trjapitzini* Myartseva and Sugonjaev, 1977**
Host: Unknown from Iran.
Iranian records: Iran (Myartseva and Sugonjaev 1977; Trjapitzin 1989).

**Genus *Epitetracnemus* Girault, 1915 *Epitetracnemus intersectus* (Fonscolombe, 1832)**
Synonym in Iranian literature: *Anabrolepis zetterstedtii* (Westwood, 1837) (Chodjai 1989; Yazdani and Rajabi 1993; Moddarres Awal 1997).
Hosts: Soft scale (Hemiptera: Coccidae) on prune tree, Soft scale on *P. vera* L. (Anacardiaceae) (Yazdani and Rajabi 1993).
Iranian records: Tehran province (Chodjai 1989; Moddarres Awal 1997) Kerman province (Yazdani and Rajabi 1993; Moddarres Awal 1997).

**Genus *Eupoecilopoda* Novicky and Hoffer, 1953 *Eupoecilopoda perpunctata* (Masi, 1942)**
Host: Chrysopidae (Neuroptera) on *P. vera* L. (Anacardiaceae) (Yazdani and Mehrnejad, 1993).
Iranian record: Kerman province (Yazdani and Mehrnejad 1993).

Genus *Gyranusoidea* Compere, 1947
*Gyranusoidea indica Shafee, Alam and Agarwal, 1975
Material examined: 3♀, 1♂, Jahrom, Fars province, ex *M. hirsutus* (Green) (Hemiptera: Pseudococcidae) on *M. alba* L. (Moraceae), 22.VII.2005, M. Fallahzadeh. Voucher specimens housed in EBRC.

**Figure 1–4.**
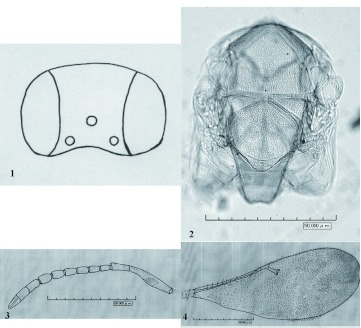
*Gyranusoidea iranica* Japoshvili and Fallahzadeh sp. nov female 1. Head; 2. Thorax; 3. Antenna; 4. Forewing. High quality figures are available online.

***Gyranusoidea iranica* Japoshvili and Fallahzadeh sp. nov. (Figures 1, 2, 3, 4)**
Female: Length (0.9–1.1 mm).
Female holotype: Length 1.013 mm. Head, thorax, abdomen and legs all yellow, except mesoscutum and scutellum with hardly noticeable narrow perpendicular brown band. Head more or less regularly reticulate on frontovertex. Scape little more than 4.7x as long as wide, in the middle with brown band which is 4.43x as wide as scape length. Ocelli forming obtuse angle. Pedicel 2.35x, F_1_- 1.75x, F_2_ - 1.65x, F_3_ - 1.67x, F_4_ - 1.67x, F_5_ - 1.64x , F_6_ - 1.63x, Clava - 4.8x as long as wide, respectively. Eye 1.76x as long as malar space. Relative measurements (holotype): HW 341.5; HH 295.8; FV 152.5; POL 51.5; OOL 23; OCL 15.3; AOL 45.8; OD 32.6 EL 187.5; EW 105; MS 122.4.
Mesoscutum and scutellum somewhat irregularly, not polygonally reticulate. Mesoscutum almost 2x as wide as long. Scutellum 1.37 as wide as long. Thorax 0.6x as long as gaster. Postmarginal: marginal:stigmal veins as 1.5:6:8.2.
Female paratype (slide-mounted). Relative measurement: SL 28.5; SW 6; FWL 139.3; FWW 55.4; MF 2.5; OL 44; GL 10.7.
Male: Color as in female, only antennae and genitalia different. Material examined: Holotype: ♀, Iran, Fars province, Beyza, ex *Chorizococcus* sp. (Hemiptera: Pseudococcidae) on grape, September 2005, M. Fallahzadeh. Paratypes: 11♀, 2♂ same data as holotype. Holotype and paratypes in EBRC.
Comments: *G. iranica* is similar to *flava* but they can be separated using the characters given in Table 1.

**Genus *Habrolepis* Förster, 1856 *Habrolepis dalmanni* (Westwood, 1837)**
Host: *Melanaspis inopinata* (Leonardi) (Hemiptera: Diaspididae) on *P. vera* L. (Anacardiaceae) (Yazdani and Rajabi, 1993).
Iranian record: Kerman province (Yazdani and Rajabi 1993).

***Habrolepis pascuorum* Mercet, 1921**
Host: *Parlatoria oleae* (Colvée) (Hemiptera: Diaspididae) on Rosaceous fruit trees (Rosaceae) (Radjabi 1989).
Iranian records: Tehran, Markazi provinces (Radjabi 1989; Moddarres Awal 1997).

***Habrolepis tergrigorianae* Trjapitzin, 1962**
Hosts: *D. prunorum* (Laing) (Hemiptera: Diaspididae) and *E. coryli* [*E. tiliae* (Linnaeus)] (Hemiptera: Coccidae) on Rosaceous fruit trees (Rosaceae) (Dastghyb Beheshti et al. 1988; Radjabi 1989; Behdad 1991; Moddarres Awal 1997; Fry 1989).
Iranian records: Isfahan province (Dastghyb Beheshti et al. 1988; Radjabi 1989; Behdad 1991; Moddarres Awal 1997), Iran (Fry 1989).

**Genus *Homalotylus* Mayr, 1876 *Homalotylus albiclavatus* (Agarwal, 1970)**
Host: Lady beetle larvae (Coleoptera: Coccinellidae) on *Citrus* (Rutaceae) and *Morus* (Moraceae) (Novin 2000) associated with *N. viridis* (Hemiptera: Pseudococcidae).
Iranian record: Khuzestan province (Novin 2000).

***Homalotylus ephippium* (Ruschka, 1923)**
Host: *Exochomus quadripustulatus* (L.) (Coleoptera: Coccinellidae) cypress tree (Cupressaceae) associated with *P. vovae* (Nasonov) (Hemiptera, Pseudococcidae) (Xu and Lotfalizadeh 2000; Lotfalizadeh and Ahmadi 2000).
Iranian records: Fars province (Xu and Lotfalizadeh 2000; Lotfalizadeh and Ahmadi 2000)

***Homalotylus eytelweinii* (Ratzeburg, 1844)**
Host: Lady beetle larvae (Coleoptera: Coccinellidae).
Iranian record: Karaj, Khuzestan province (Japoshvili and Noyes 2005).

***Homalotylus flaminius* (Dalman, 1820)**
Host: *Nephus bipunctatus* (Kugelann) (Coleoptera: Coccinellidae) on *Lactuca serriola* L. (Asteraceae) associated with *P. kimmericus* (Kiritshenko) (Hemiptera: Pseudococcidae) (Fallahzadeh et al. 2006a).
Iranian records: Fars province (Fallahzadeh et al. 2006a).

***Homalotylus nigricornis* Mercet, 1921**
Host: *Scymnus* sp. (Coloptera: Coccinellidae) associated with *Aphis gossypii* (Glover) (Hemiptera: Aphididae) (Lotfalizadeh and Ebrahimi 2001).
Iranian record: Ardabil province (Lotfalizadeh and Ebrahimi 2001).

***Homalotylus quaylei* Timberlake, 1919**
Hosts: *Nephus includens* Kirsch (Coleoptera: Coccinellidae) associated with *N. viridis* (Newstead) (Hemiptera: Pseudococcidae) (Asadeh and Mossadegh 1991; Novin 2000; Novin et al. 2000), *Scymnus subvillosus* (Goeze) on *Citrus* associated with *P. citri* (Risso) (Hemiptera: Pseudococcidae) (Maafi et al. 1998).
Iranian records: Iran (OILB 1971), Khuzestan province (Asadeh and Mossadegh 1991; Novin et al. 2000), Mazandaran province (Maafi et al. 1998).

**Table: 1.**
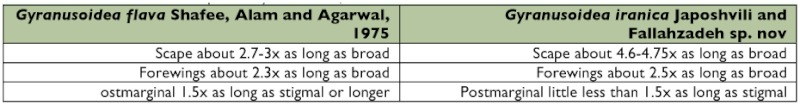
Characters used to separate *Gyranusoidea flava* and *G. iranica*.

***Homalotylus sinensis* Xu and He, 1997**
Hosts: *N. bipunctatus* (Kugelann) (Coleoptera: Coccinellidae) on *L. serriola* L. (Asteraceae) associated with *P. kimmericus* (Kiritshenko) (Hemiptera: Pseudococcidae) (Fallahzadeh et al. 2006a), *Exochomus nigromaculatus* (Goeze) and *E. quadripustulatus* (L.) (Coleoptera: Coccinellidae) on cypress tree (Cupressaceae) associated with *P. vovae* (Nasonov) (Hemiptera: Pseudococcidae) (Ameri et al. 2007; Talebi et al. 2008).
Iranian records: Fars province (Fallahzadeh et al. 2006a; Ameri et al. 2007; Talebi et al. 2008).

***Homalotylus turkmenicus* Myartseva, 1981**
Host: Unknown from Iran.
Iranian record: Iran (Trjapitzin 1989).
Material examined: 1♀, 2♂, Jahrom, Fars province, 28°31′N, 53°34′E, 1280m, ex **E. quadripustulatus (L.) (Coleoptera: Coccinellidae) on grape, 2. V. 2007, M. Fallahzadeh.

*Homalotylus vicinus Silvestri, 1915
Material examined: 1♀, 2♂, Jahrom, Fars province, 28°31′N, 53°34′E, 1280m, ex **N. bipunctatus Kugelann (Coloeptera: Coccinellidae) on grape, 5.VI.2007, M. Fallahzadeh. Voucher specimens housed in EBRC.

**Genus *Isodromus* Howard, 1887 *Isodromus atriventris* Ashmead, 1900**
Hosts: *C. carnea* Stephens and *S. fedtchenkoi* (McLachlan) (Neuroptera: Chrysopiodae) on cypress tree (Cupressaceae) associated with *P. vovae* (Nasonov) (Hemiptera: Pseudococcidae) (Xu and Lotfalizadeh 2000, Lotfalizadeh and Ahmadi 2000; Lotfalizadeh, 2000a).
Iranian records: Fars province (Xu and Lotfalizadeh 2000; Lotfalizadeh and Ahmadi 2000; Lotfalizadeh 2000a).

***Isodromus collimaculatus* Xu and Lotfalizadeh, 2000**
Hosts: *C. carnea* Stephens and *S. fedtchenkoi* (McLachlan) (Neuroptera: Chrysopidae) on cypress tree (Cupressaceae) associated with *P. vovae* (Nasonov) (Hemiptera: Pseudococcidae) (Xu and Lotfalizadeh 2000, Lotfalizadeh and Ahmadi 2000; Lotfalizadeh 2000a).
Iranian records: Fars province (Xu and Lotfalizadeh 2000; Lotfalizadeh 2000a).

Genus *Leptomastidea* Mercet, 1916
*Leptomastidea abnormis (Girault, 1915)
Material examined: 2♂, 2♀, Meymand, Fars province, ex *Planococcus ficus* (Signoret) (Hemiptera: Pseudococcidae) on grape, 26. VI. 2005, M. Fallahzadeh. Voucher specimens housed in EBRC.

***Leptomastidea alleni* Noyes and Hayat, 1994**
Hosts: *N. viridis* (Newstead) (Hemiptera: Pseudococcidae) on *Citrus sinensis* (L.), *C. aurantium* (L.) (Rutaceae) and *Althaea* sp. (Malvaceae), *P. vovae* (Nasonov) (Hemiptera: Pseudococcidae) on *Cupressus* sp. (Cupressaceae) (Fallahzadeh et al., 2008).
Iranian record: Fars province (Fallahzadeh et al. 2008).

**Genus *Leptomastix* Förster, 1856 *Leptomastix flava* Mercet, 1921**
Synonym in Iranian literature: *Leptomastix flavus* Mercet, 1921 (Kiriukhin 1947; Radjabi 1989; Behdad 1991; Moddarres Awal 1997).
Hosts: *P. aceris* (Signoret) (Hemiptera: Pseudococcidae) (Kiriukhin 1947) *P. aceris *(Hemiptera: Pseudococcidae) on Roseous fruit trees (Rosaceae) (Radjabi 1989), *Eulecanium rugulosum* (Archangelskaya) (Hemiptera: Coccidae) on *P. vera* L. (Anacardiceae) (Yazdani and Rajabi, 1993).
Iranian records: Iran (Kiriukhin 1947), Isfahan, Tehran provinces (Radjabi 1989; Behdad 1991; Moddarres Awal 1997), Kerman province (Yazdani and Rajabi 1993).
Material examined: 1♀, 1♂, Jahrom, Fars province, 28°31′N, 53°34′E, 1280m, ex *P. ficus* (Signoret) (Hemiptera: Pseudociccidae) on grape, 15.V.2007, M. Fallahzadeh.

*Leptomastix dactylopii Howard, 1885
Material examined: 2♀, 1♂, Jahrom, Fars province, 28°31′N, 53°34′E, 1280m, ex *P. ficus* (Signoret) (Hemiptera: Pseudococcidae) on grape, 13.VI.2007, M. Fallahzadeh. Voucher specimens housed in EBRC.

***Leptomastix histrio* Mayr, 1876**
Host: *P. kimmericus* (Kiritshenko) (Hemiptera: Pseudococcidae) on *L. serriola* L. (Asteraceae) (Fallahzadeh et al. 2006b).
Iranian record: Fars province (Fallahzadeh et al. 2006b).

**Genus *Metaphycus* Mercet, 1917 *Metaphycus angustifrons* Compere, 1957**
Host: *C. hesperidum* Linnaeus (Hemiptera: Coccidae) on *Robinia* (Papilionaceae), *Morus* (Moraceae), *Ailanthus* (Simaroubaceae), *Diospyros* (Ebenaceae) (Davoodi et al. 2003a,c; 2004d).
Iranian records: Tehran province (Davoodi et al. 2003a, c; 2004d).

***Metaphycus anneckei* Guerrieri and Noyes, 2000**
Host: Unknown from Iran.
Iranian record: Iran (Guerrieri and Noyes 2000).

***Metaphycus claviger* (Timberlake, 1916)**
Host: *C. hesperidum* Linneaus (Hemiptera: Coccidae) on *Citrus* (Rutaceae) and apple tree (Rosaceae) (Davoodi et al. 2004a, b).
Iranian records: Fars province (Davoodi et al. 2004a, b).

***Metaphycus flavus* (Howard, 1881)**
Host: *C. hesperidum* Linneaus (Hemiptera: Coccidae) (Herting 1972).
Iranian records: Iran (Herting, 1972; Guerrieri and Noyes 2000).

***Metaphycus helvolus* (Compere, 1926)**
Host: *Saissetia oleae* (Olivier) (Hemiptera: Coccidae) (Esmaili 1983; Moddarres Awal 1997).
Iranian records: Caspian sea area (Esmaili 1983; Moddarres Awal 1997), Iran (Prinsloo 1984; Trjapitzin 1989; Noyes and Hayat 1994).

***Metaphycus hodzhevanishvilii* Yasnosh, 1972**
Host: *Stotzia ephedrae* (Newstead) (Hemiptera: Coccidae) on *Ephedra procera* Fisch. Et Mey (Ephedraceae) (Hesami et al. 2007).
Iranian record: Fars province (Hesami et al. 2007).

***Metaphycus lounsburyi* (Howard, 1898)**
Host: *S. oleae* (Olivier) (Hemiptera: Coccidae) (Esmaili 1983; Moddarres Awal 1997).
Iranian records: Caspian sea area (Esmaili 1983; Moddarres Awal 1997), Iran (Noyes and Hayat 1994).

***Metaphycus pulvinariae* (Howard, 1881)**
Host: *Didesmococcus unifasciatus* (Archangelskeya) on *Amygdalus* (Rosaceae) (Davoodi et al. 2004a).
Iranian record: East Azerbaijan (Davoodi et al. 2004a).

***Metaphycus punctipes* (Dalman, 1820)**
Hosts: *E. coryli* [*E. tiliae* (Linnaeus)] (Hemiptera: Coccidae) on quince tree (Rosaceae) (Ebrahimi 1993), *S. prunastri* (Boyer de Fonscolombe) (Hemiptera: Coccidae) on *Amygdalus* (Rosaceae) (Esfandiari et al. 2005).
Iranian records: Iran (OILB 1971), Chaharmahal-Bakhtiyari province (Ebrahimi 1993; Esfandiari et al. 2005).

***Metaphycus stanleyi* Compere, 1940**
Host: *S. oleae* (Olivier) (Hemiptera: Coccidae) (Esmaili 1983; Moddarres Awal 1997).
Iranian records: Caspian sea area (Esmaili 1983; Moddarres Awal 1997).

***Metaphycus zebratus* (Mercet, 1917)**
Host: *Parthenolecanium* sp. (Hemiptera: Coccidae).
Iranian record: Iran (OILB 1971).

**Genus *Microterys* Thomson, 1876 *Microterys hortulanus* Erdös, 1956**
Hosts: *S. prunastri* (Boyer de Fonscolombe) (Hemiptera: Coccidae) on plum tree (Rosaceae), *D. unifasciatus* (Archangelskaya) (Hemiptera: Coccidae) on *Amygdalus* (Rosaceae), *E. coryli* [*E. tiliae* (Linnaeus)] (Hemiptera: Coccidae) on plum tree (Rosaceae) (Davoodi et al. 2002, 2004d).
Iranian records: East Azerbaijan and Tehran provinces (Davoodi et al. 2002; 2004d).

**Figure 5–9.**
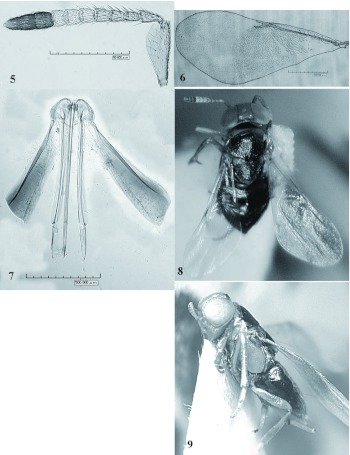
*Microterys iranica* Japoshvili and Fallahzadeh sp. nov. female 5. Antenna; 6. Forewing; 7. Ovipositor; 8. Body view from dorsal side; 9. Body view from side view. High quality figures are available online.

***Microterys iranicus* Japoshvili and Fallahzadeh sp. nov. (Figures 5, 6,7, 8, 9)**
Female holotype. Length 2.3 mm. Head yellow, except brown band across vertex back margin behind posterior ocelli. Eyes violet. Scape, pedicel, and flagellar segments 1–3 yellow, F_4–6_ white, clava brown. Pronotum, mesopleuron, and axilla yellow, only area where axillae join brown. Mesoscutum with green-silver and scutellum with violet-silver, almost black, metallic reflection. Propodeum, metanotum, and abdomen brown, with some violetsilver metallic reflections. Legs yellow, hind coxa brown.
  Head about 1.4x as wide as high and about 3.67x as wide as FV; FV in dorsal view almost 2x as long as wide. OOL almost 2.3x shorter then OCL and POD. Ocelli forming equilateral triangle. Scape about 3x as long as wide. Pedicel 1.8x as long as wide; F_1_ 0.57x as long as pedicel and 1.2x as long as wide. F_2_ 1.24x as long as F_1_. F_2_ - 1.44x, F_3_ - 1.15x, F_4_ -1.07x, F_5_ - 1, F_6_ - 0.9x as long as wide respectively. The longest segment is F_2_ and shortest F_1_. Toruli separated from each other by 1.56x and from clypeal margin 1.17x their maximum diameter. Upper margin of toruli in same line as lowest eye margin. Relative measurement (holotype): HW 399.8; FV 107.5; OD 24; POL 8.1; OOL 52.5; OCL 26.4; AOL 45; MS 136.4; EL 213.7; 135.7. Mesoscutum and scutellum flat, of equal length, scutellum as long as wide, mesoscutum 1.35x as wide as long. Fore wing 2.3x as long as wide. Submarginal vein with 17 setae. Submarginal, marginal, postmarginal and stigmal veins as 9.6:1.34:1:1.4. Band on the forewing not clear, wings infuscate on basal 0.64x, hyaline apical part 0.36x as long as wing. Midtibial spur 0.84x as long as basitarsus. Female paratype (slide-mounted). Relative measurements: SL 42; SW 15.3; FWL 295.5; FWW 132.6; OL 80.5; GL 17.8; OPL 60.5; OPW 17.2.
Male: Head, mesoscutum, scutellum black with green-golden metallic reflection. Antenna yellow, pedicel brown dorsally. Tegula and mesopleuron yellow. Pedicel more then 3 times shorter that of F1. Abdomen, metanotum and propodeum brown with green-violet metallic reflection. Material examined: Holotype: ♀, Iran, Fars province, Mian-Jangle, Fasa, 22°12'N, 53°23'E, 1680m, ex *Sphaerolecanium prunastri* (Hemiptera: Coccidae), on *Prunus scoparia*, 2.VII.2007, M. Fallahzadeh. Paratypes: 2♀, 1♂ same data as holotype. Holotype and paratypes in EBRC.
Comments: *Microterys iranicus* is most to close to *M. darevskii* Trjapitzin, 1968 but they can be separated using the characters given in Table 2.

***Microterys lunatus* (Dalman, 1820)**
Host: *E. coryli* [*Eulecanium tiliae *(Linnaeus)] (Hemiptera: Coccidae) on *Malus* (Rosaceae) (Chodjai 1968; Chodjai 1989).
Iranian records: Fars province (Chodjai 1968; Chodjai 1989; Ebrahimi 1993; Moddarres Awal 1997), Iran (OILB 1971).

***Microterys masii* Silvestri, 1919**
Host: *Parthenolecanium* sp. (Hemiptera: Coccidae) (OILB 1971).
Iranian record: Iran (OILB 1971).

***Microterys nietneri* (Motschulsky, 1859)**
Host: *C. hesperidum* L. (Hemiptera: Coccidae) (Farahbakhsh 1961; Davoodi et al. 2004d).
Iranian records: Mazandaran province (Farahbakhsh 1961), Tehran province (Davoodi et al. 2004d).

***Microterys nuticaudatus* Xu, 2000**
Host: *Parthenolecanium corni* (Bouche) (Hemiptera: Coccidae) on *Morus* (Moraceae) (Davoodi et al. 2003b, 2004a).
Iranian records: East Azerbaijan and Tehran provinces (Davoodi et al. 2003b, 2004a).

**Genus *Ooencyrtus* Ashmead, 1900 *Ooencyrtus cinctus* Prinsloo, 1987**
Host: *Bucculatrix ulmella* Zeller (Lepidoptera: Bucculatricidae) on elm tree (Ulmaceae) (Baniameri and Mohammadi- Pour 2007).
Iranian record: Tehran province (Baniameri and Mohammadi-Pour 2007).

***Ooencyrtus kuvanae* (Howard, 1910)**
Hosts: *C. carnea* Stephens and *S. fedtchenkoi* (McLachlan) (Neuroptera: Chrysopiodae) on cypress tree (Cupressaceae) (Xu and Lotfalizadeh 2000; Lotfalizadeh and Ahmadi 2000; Lotfalizadeh 2000a).
Iranian records: Fars province (Xu and Lotfalizadeh 2000, Lotfalizadeh and Ahmadi 2000; Lotfalizadeh 2000a).

***Ooencyrtus masii* (Mercet, 1921)**
Host: *Ocneria terebinthina* Stgr. (Lepidoptera: Lymantriidae) on wild pistachio trees (Anacardiaceae) (Sabahi et al. 1998, 1999; Omid et al. 2005).
Iranian records: Fars province as *Ooencyrtus* cf *masii* (Sabahi et al. 1998, 1999), Khojir protected area (Omid et al. 2005).

***Ooencyrtus nigerrimus* Ferrière and Voegelé, 1961**
Host: *Eurygaster integriceps* Puton (Hemiptera: Scutelleridae) (Radjabi and Amir- Nazari 1989; Moddarres Awal, 1997).
Iranian records: Tehran, Hamadan, Lorestan, Markazi provinces (Radjabi and Amir- Nazari 1989; Moddarres Awal 1997).

***Ooencyrtus telenomicida* (Vassiliev, 1904)**
Hosts: *Aelia* sp. (Hemiptera: Pentatomodae), *Eurygaster* sp. (Scutelleridae) on *Triticum aestivum* L. (Gramineae) (Chodjai 1968; Chodjai 1989; Davatchi and Chodjai 1969), *E. integriceps *Puton, *Aelia* sp., *Dolycoris bacarum* (L.), *Carpocoris fuscipinus* (Boheman) and *Macrocerus marginatus* (Hemeiptera: Pentatomidae) on cereal fields (Gramineae) (Radjabi and Amir-Nazari 1989; Moddarres Awal 1997), *E. integriceps* (Iranipour et al. 1998), *Acrosternum* spp. and *Brachynema* spp. on *P. vera* L. (Anacardiaceae) (Hashemi Rad et al. 2002), *E. integriceps* on cereal fields (Gramineae).
Iranian records: Tehran, Hamadan, Lorestan, Mazandaran, Markazi provinces (Chodjai 1968; Chodjai 1989; Davatchi and Chodjai 1969; Radjabi and Amir-Nazari 1989; Moddarres Awal 1997); East Azerbaijan (Iranipour et al. 1998), Kerman province (Hashemi Rad et al. 2002), Qazvin province (Noori et al. 2003), Iran (Herting 1971; Trjapitzin 1989; Zhang and Huang 2005).

Genus *Paranathrix* Myartseva, 1980
*Paranathrix tachikawai (Shafee, Alam and Agarwal, 1975)
Material examined: 6♀, 1♂, Minab, Hormozgan province, ex **Ferrisia virgata (Ceckerell) (Hemiptera: Pseudococcidae) on *Althea* 9.X.2007, M. Fallahzadeh. Voucher specimens housed in EBRC.

**Table 2.**
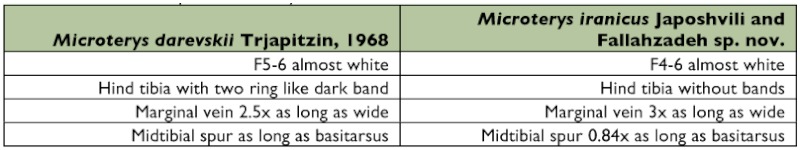
Characters used to separate *Microterys darevskii* and *M. iranicus*.

**Genus *Paraphaenodiscus* Girault, 1915 *Paraphaenodiscus sugonjaevi* Myartseva, 1980**
Host: Unknown from Iran.
Iranian records: Iran (Myartseva 1980; Trjapitzin 1989).

**Genus *Prionomitus* Mayr, 1876 *Prionomitus mitratus* (Dalman, 1820)**
Host: *A. pistaciae* (Burckhardt and Lauterer) (Hemiptera: Psyllidae) on *P. vera *L. (Anacardiaceae) (Dezianian and Sahragard 2000; 2004).
Iranian record: Semnan province as *Prionomitus* near *mitratus* (Dezianian and Sahragard 2000; 2004).

**Genus *Prochiloneurus* Silvestri, 1915 *Prochiloneurus aegyptiacus* (Mercet, 1929)**
Hosts: *H. quaylei* Timberlake (Hymenoptera: Encyrtidae) (OILB 1971), Parasitoid of *Anagyrus* spp. (Hymenoptera: Encyrtidae) (Hesami and Fallahzadeh 2004), Hyperparasitoid of *M. hirsutus* (Green) (Hemiptera: Pseudococcidae) on *M. alba* L. (Moraceae) (Fallahzadeh et al. 2007).
Iranian records: Iran (OILB 1971), Fars province (Hesami and Fallahzadeh 2004; Fallahzadeh et al. 2007).

*Prochiloneurus bolivari Mercet, 1919
Material examined: 2♀, Jahrom, Fars province, 28°31′N, 53°34′E, 1280m, ex *P. ficus* (Signoret, 1875) (Hemiptera: Pseudococcidae) on grape, 19.VII.2007, M. Fallahzadeh. Voucher specimens housed in EBRC.

***Prochiloneurus indicus* Shafee, Alam and Agarwal, 1975**
Host: Hyperparasitoid *N. viridis* (Newstead) (Hemiptera: Pseudococcidae) (Asadeh and Mossadegh 1991) on *Citrus* (Rutaceae) and *M. alba* L. (Moraceae).
Iranian record: Khuzestan province (Asadeh and Mossadegh 1991).

***Prochiloneurus pulchellus* Silvestri, 1915**
Host: *Pseudococcus* on *Tamarix *(Tamaricaceae) (Japoshvili and Noyes 2005).
Iranian record: Khuzestan province (Japoshvili and Noyes 2005).

**Genus *Psyllaephagus* Ashmead, 1900 *Psyllaephagus claripes* Trjapitzin, 1967**
Host: *Psyllopsis repens* Loginova (Hemiptera: Psyllidae) on *Fraxinus* (Oleaceae) (Rajabi Mazhar et al. 2004; Rajabi Mazhar and Sadeghi 2006).
Iranian records: Hamadan province (Rajabi Mazhar et al. 2004; Rajabi Mazhar and Sadeghi 2006).

***Psyllaephagus pistaciae* Ferrière, 1961**
Host: *Agonoscena pistaciae* (Burckhardt & Lauterer) (Hemiptera: Psyllidae) on *P. vera* L. (Anacardiaceae) (Ferrière 1961; Ebrahimi et al. 1998; Emami and Mehrnejad 2002, 2004, 2006; Mehrnejad 2002a,b,c, 2008; Mehrnejad and Copland 2006, 2007).
Iranian records: Iran (Ferrière 1961; Herting 1972; Trjapitzin 1989), Kerman province (Mehrnejad et al. 1995; Ebrahimi et al. 1998; Emami and Mehrnejad 2002; Emami and Mehrnejad 2004, 2006; Mehrnejad 2002a,b,c, 2008; Mehrnejad and Copland 2006, 2007).

***Psyllaephagus stenopsyllae* (Tachikawa, 1963)**
Host: *Diaphorina citri* Kuwayama (Hemiptra: Psyllidae) on *Citrus* (Rutaceae).
Iranian record: Hormozghan province (Ameri et al. 2006).

***Psyllaephagus zdeneki* Noyes and Fallahzadeh, 2005**
Host: *Euphyllura pakistanica* Loginova, 1973 (Hemiptera: Psyllidae) on *Oleae europea* L. (Oleaceae) (Noyes and Fallahzadeh 2005).
Iranian record: Fars province (Noyes and Fallahzadeh 2005).

**Genus *Syrphophagus* Ashmead, 1900 *Syrphophagus aeruginosus* (Dalman, 1820)**
Hosts: *Sphaerophoria* spp., *Eopodes corollae* (Fab.) and *Episyrphus balteatus* (DeGeer) (Diptera: Syrphidae) (Xu and Lotfalizadeh 2000; Lotfalizadeh 2000b).
Iranian records: East Azerbaijan Province (Xu and Lotfalizadeh 2000; Lotfalizadeh 2000b).

***Syrphophagus aphidivorus* (Mayr, 1876)**
Hosts: Hyperparasitoid of *Agonoscena pistaciae* (Burckhardt and Lauterer) (Hemiptera: Psyllidae) on *P. vera* L. (Anacardiaceae) (Yazdani and Mehrnejad 1993; Ebrahimi et al. 1998; Mehrnejad 2000b; Emami and Mehjrnejad 2002, 2004, 2006; Mehrnejad and Emami 2005), Hyperparasitoid of *Aphis craccivora* Koch., *A. gossypii* (Glover), (Hemiptera: Aphididae) on *Alhagi camlorum* Fisch. and *Glycyrrhiza glabra* L. (Papilionaceae) in pistachio orchards (Emami and Mehjrnejad 2004, 2006; Mehrnejad and Emami 2005), Hyperparasitoid of *Chromaphis juglandicola* (Hemiptera: Aphididae) (Rakhshani et al. 2004) Hyperparasitoid of *Pauesia antennata* (Mukerji) (Hymenoptera: Braconidae: Aphidiinae) (Rakhshani et al. 2005).
Iranian records: Kerman province (Yazdani and Mehrnejad 1993; Ebrahimi et al. 1998; Mehrnejad 2000b; Emami and Mehjrnejad 2002, 2004, 2006; Mehrnejad and Emami 2005); Tehran province (Rakhshani et al. 2004); Sistan ans Baluchastan province (Rakhshani et al. 2005); Iran (Japoshvili, Abrantes 2006).

**Genus *Trechnites* Thomson, 1876 *Trechnites insidiosus* (Crawford, 1910)**
Host: *Psylla pyricola* [*Cacopsylla pyricola *(Foerster)] (Hemiptera: Psyllidae) (Behdad 1991)
Iranian record: Tehran province (Behdad 1991).

**Genus *Zaomma* Ashmead, 1900 *Zaomma lambinus* (Walker, 1838)**
Synonyms in ranian literatures: *Apterencyrtus microphagus* (Mayr 1876), *Chiloneurus microphagus* Mayr, 1876, *Chiloneurinus microphagus* (Mayr 1876) (Chodjai 1989; Radjabi 1989; Moddarres Awal 1997).
Hosts: *Lepidosaphes malicola* Borchsenius (Hemiptera: Diaspididae) on Roseous fruit trees (Rosaceae) (Chodjai 1989; Radjabi 1989; Moddarres Awal 1997), *Tecaspis asiatica* [*Chlidaspis asiatica* (Archangelskaya)] (Hemiptera: Diaspididae) on Roseous fruit trees (Rosaceae) (Radjabi 1989); *Lepidosaphes pistaciae* Archangelskaya (Hemiptera: Diaspididae) on *P. vera* L. (Anacardiaceae) (Mansouri 2005).
Iranian records: Tehran province (Chodjai 1989; Moddarres Awal 1997), Chahar Mahalo/ Bakhtiari, Esfahan, Fars, Markazi, Tehran, Semnan provinces (Radjabi 1989; Moddarres Awal 1997), Esfahan province (Mansouri 2005).

## Abbreviations

AOL: distance between posterior and anterior ocelli;
EL: maximum eye length;
FV: minimum frontovertex width;
F1, F2, etc.: first funicle segment, second funicle segment, etc.;
GL: maximum length of gonostylus (= third valvula);
HW: maximum head width;
MS: malar space (shortest distance from eye to mouth margin);
OCL: occipital ocellar line (distance of posterior ocellus from occipital margin);
OD: greatest diameter of an ocellus;
OL: ovipositor length;
OOL: ocular-ocellar line (shortest distance between posterior ocellus and adjacent eye margin);
OPL: length of outer plate of ovipositor;
OPW: maximum width of outer plate of ovipositor;
POL: posterior ocellar line (= the shortest distance between the posterior ocelli);
SL: scape length;
SW: scape width;
Acronym for depository, EBRC: Insect collection of Entomology and Biocontrol Research Centre, Ilia State University, Tbilisi, Georgia
